# Dermatologic Toxicities in Epidermal Growth Factor Receptor and Multikinase Inhibitors

**DOI:** 10.6004/jadpro.2012.3.3.2

**Published:** 2012-05-01

**Authors:** Beth Eaby-Sandy, Carolyn Grande, Pamela Hallquist Viale

**Affiliations:** From Abramson Cancer Center, Philadelphia, Pennsylvania, and Goleta, California

## Abstract

Targeted therapies have produced significant treatment advances for patients diagnosed with a variety of tumor types. These therapies are associated with unique dermatologic toxicities that may hamper treatment efforts and cause significant discomfort for patients. Prevention and management of these toxicities can allow patients to remain on therapy and hence receive maximum clinical benefit from the drug.

Treatment of many types of tumors has evolved from chemotherapy to a more personalized approach, using strategies that can target receptors associated with the specific cancer cell. Over the past decade, numerous targeted agents have been approved for use by the US Food and Drug Administration (FDA). There are two types of targeted therapies that can cause significant dermatologic toxicities: epidermal growth factor receptor (EGFR) inhibitors and multikinase inhibitors.

There have been very few randomized clinical trials addressing the management of dermatologic toxicities for the EGFR and multikinase inhibitors, mostly due to difficult study designs, a lack of validated tools for assessment, and low patient enrollment (Burtness et al., 2009; Lacouture et al., 2011). The dermatologic side effects can be uncomfortable, and at times disfiguring, to patients, sometimes requiring dose reductions or discontinuation of therapy. This article will address the causes of these side effects, assessment methods, and strategies for prevention and management.

## Pathophysiology and Rationale for Use in Cancer Therapy

Most malignant tumors have multiple signaling pathways that enable their growth and progression. This can lead to dysregulated cell signaling, inhibition of programmed cell death, and growth and spread of disease. The epidermal growth factor receptor, also known as HER1, is a part of the human epidermal growth factor receptor (HER) family, which also includes HER2/*neu*, HER3, and HER4 (Lynch et al., 2007). Ligands, proteins that are growth factors, activate EGFR by binding to the receptor. Once bound, the two receptors dimerize (with either a second EGFR or another member of the HER family); EGFR paired with EGFR is an example of homodimerization, and EGFR paired with HER2 is representative of heterodimerization (Oishi, 2008). The act of dimerization then initiates the activation of tyrosine kinase (TK) activity and the subsequent initiation of the downstream signaling pathways that are involved in tumor cell proliferation, migration, adhesion, and angiogenesis and play a role in the inhibition of cell apoptosis (Lynch et al., 2007).

Epidermal growth factor receptor is overexpressed in many different malignancies. Because EGFR is abnormal in many tumor types and overexpression is critical to disease progression and poor prognosis, inhibition of the receptor with a pharmacologic agent is an important part of cancer therapy (Lynch et al., 2007). However, the EGFR is also important in normal skin development and disproportionately affected when inhibitors of the receptor are administered in cancer therapy. Epidermal growth factor receptor is highly expressed in the epidermal keratinocytes, the sebaceous glands, and the epithelium of the hair follicle; therefore, EGFR inhibitor therapy can produce several dermatologic adverse events (Eaby, Culkin, & Lacouture, 2008).

In addition to EGFR, there are multiple other growth factors and cytokines that play a role in cell growth and differentiation. They can be either receptor or nonreceptor tyrosine kinases. The distinction between them is that regulatory activity aiding in cell growth, activation, and differentiation can be either extracellular or intracellular (Adjei & Hidalgo, 2005). The ligand binding of receptor tyrosine kinases is extracellular, whereas the nonreceptor tyrosine kinases are activated through links to membrane receptors. In either case, when ligand binding occurs, a cascade of downstream events that can lead to abnormal cell proliferation transpires. The components of the dysregulated signaling, specific to cancer cells, can be identified as targets for new anticancer treatment (Adjei & Hidalgo, 2005).

Some of the relevant pathways being targeted for inhibition by multikinase inhibitors are as follows: Abelson murine leukemia (Abl), Rous sarcoma oncogene (Src), platelet-derived growth factor (PDGF), vascular endothelial growth factor (VEGF), fibroblast growth factor (FGF), and epidermal growth factor (EGF). There are multiple FDA-approved agents that are developed as single-target tyrosine kinase inhibitors or agents that target multiple kinase pathways for inhibition of tumor growth, progression, cell proliferation, and new blood vessel formation. These multikinase inhibitors, similar to EGFR inhibitors, are known to cause numerous dermatologic toxicities. A full list of EGFR and multikinase inhibitors, along with their specific targets, indications, and known dermatologic toxicities, can be seen in Table 1.

**Table 1:**
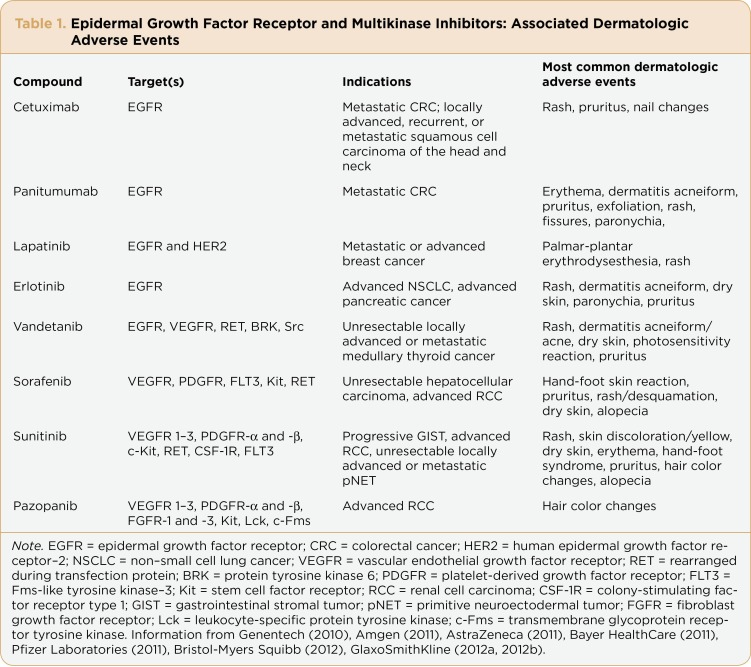
Table 1. Epidermal Growth Factor Receptor and Multikinase Inhibitors: Associated Dermatologic Adverse Events

## Dermatologic Toxicities Associated With EGFR Inhibitors

### Papulopustular Rash

**Figure 1:**
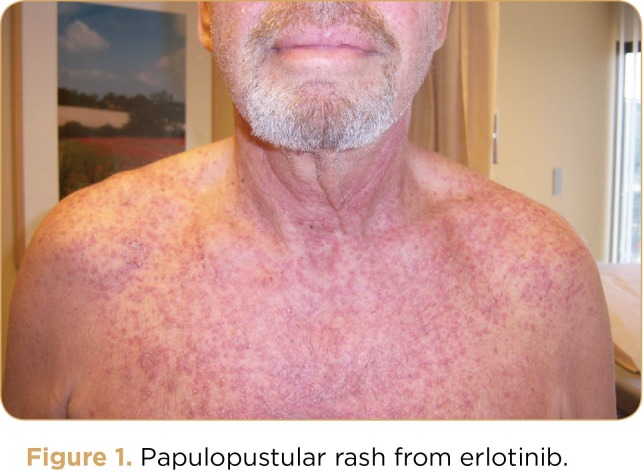
Figure 1. Papulopustular rash from erlotinib.

Although the EGFR inhibitors are associated with a variety of toxicities, including diarrhea, the most commonly seen adverse event is a papulopustular rash (see Figure 1). The pathobiology of the papulopustular rash is thought to occur due to damage to the proliferating keratinocytes found in the basal layers of the epidermis following EGFR inhibition (Fox, 2006). This subsequently leads to recruitment of inflammatory cells and macrophages, causing cutaneous injury, which results in papulopustular rash as well as periungual inflammation and xerosis (Lacouture, 2006).

Papulopustular rash appears in up to 80% to 90% of patients, with mild to moderate severity (Lacouture et al., 2011; Burtness et al., 2009; Perez-Soler et al., 2005).It is typically papulopustular in appearance and distinctly different from acne, although the word "acneiform" has been used to describe the EGFR inhibitor–associated rash (Burtness et al., 2009). The rash primarily affects the face, scalp, neck, upper chest, and back. It is associated with the papules and pustules often seen with acne vulgaris, but it lacks the comedones that are classic in acne presentation (Burtness et al., 2009). The rash generally appears around 8 to 10 days after the start of therapy with the EGFR inhibitor and peaks approximately 2 weeks after initiation; it tends to diminish after 4 to 6 weeks of being on EGFR therapy (Lynch, 2007). Although the rash is thought to occur because of inflammation due to inhibition of EGFR signaling in the skin, hair follicles, and sebaceous glands, biopsy of specific lesions has produced cultures of *Staphylococcus aureus*, suggesting that more frequent biopsies may pick up previously undetected infections (Amitay-Laish, David, & Stemmer, 2010).

The rash associated with cetuximab (Erbitux), panitumumab (Vectibix), and erlotinib (Tarceva) has been linked to a higher response rate and longer survival in published trial data, with the severity of rash related to increased survival (Burtness et al., 2009). Thus, treatment of the rash is recommended over dose reduction whenever possible (Burtness et al., 2009).

Grading of papulopustular rash and the accompanying dermatologic side effects has been a challenge. The National Cancer Institute (NCI) Common Terminology Criteria for Adverse Events (CTCAE) grading scale, which is most commonly used to grade adverse events in clinical trials, tends to serve as the cornerstone for grading toxicity. However, the most current version of this scale (CTCAE v4.03) does not offer specific EGFR inhibitor rash grading but rather a broad category called "skin and subcutaneous tissue disorders" (NCI, 2010). The Multinational Association of Supportive Care in Cancer (MASCC) Skin Toxicity Study Group has proposed a more comprehensive grading scale specific to the dermatologic adverse events associated with EGFR inhibitors (Lacouture et al., 2010a). Use and validation of this scale could offer a more detailed tool to capture and grade the severity of these dermatologic toxicities.

The papulopustular rash seen with EGFR inhibitors can have a negative effect on patients’ quality of life as well as produce uncomfortable physical symptoms (Oishi, 2008). It can be significant and potentially lead to the interruption or even discontinuation of therapy (Hassel, Kripp, Al-Batran, & Hofheinz, 2010). Therefore, optimal management of the rash is essential to avoid cessation of needed treatment. Despite the frequency of this side effect in patients receiving EGFR inhibitor therapy, definitive treatment strategies are lacking. There have been few controlled, randomized studies published offering evidence-based recommendations for management of the EGFR inhibitor–associated rash (Lacouture et al., 2011). Even in the most significant published trials examining treatment options for EGFR inhibitor–associated rash, the number of patients enrolled is below 500 (see Table 2). Clearly, there is a need for large, randomized, controlled studies examining optimal treatment strategies.

**Table 2:**
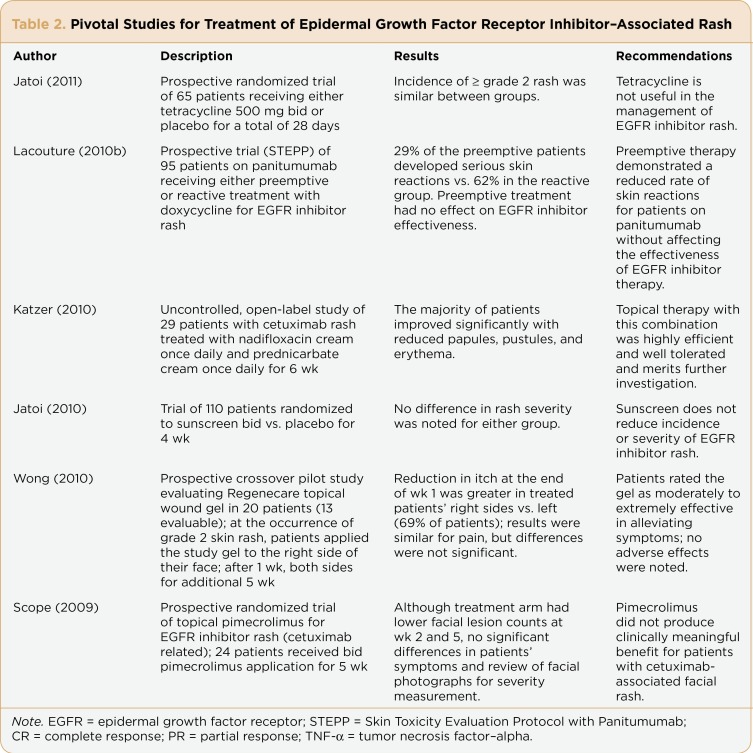
Table 2. Pivotal Studies for Treatment of Epidermal Growth Factor Receptor Inhibitor–Associated Rash

**Table 2:**
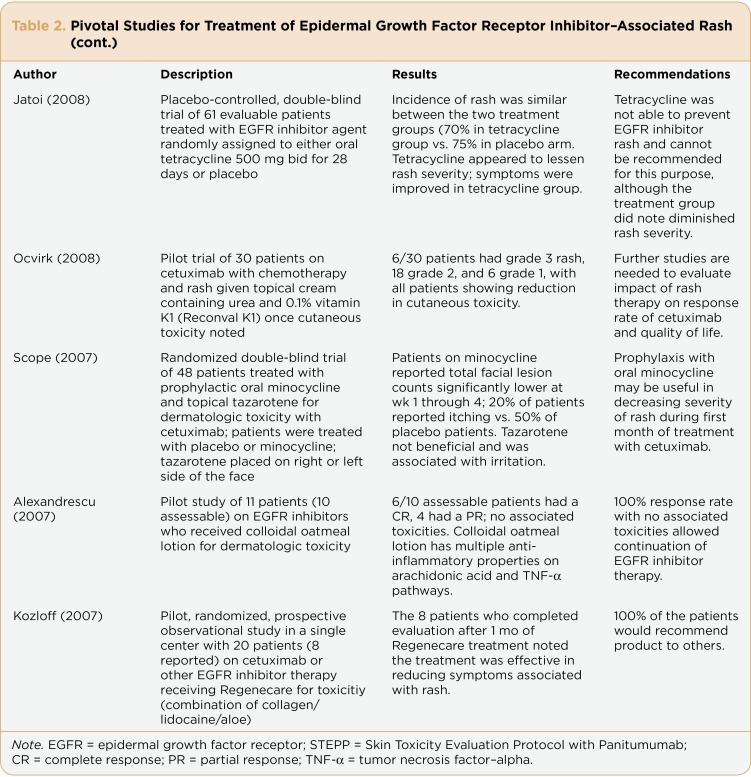
Table 2. Pivotal Studies for Treatment of Epidermal Growth Factor Receptor Inhibitor–Associated Rash (cont.)

Based on the evidence gleaned from the trials in Table 2, treatment with an oral semisynthetic tetracycline agent (not the original formulation) can be useful in an attempt to lessen the severity of EGFR inhibitor–associated rash. Tetracycline has been an established therapy for acne for over 50 years, primarily because of the drug’s anti-inflammatory and immunomodulatory properties (Ehmann, Ruzicka, & Wollenberg, 2011). However, the two negative trials by Jatoi and colleagues (2008, 2011) summarized in Table 2 demonstrate that the original formulation is not as effective as the semisynthetics; therefore, minocycline or doxycycline should be the choice for systemic antibiotic therapy. In severe cases, oral steroids can offer symptom improvement, but this is not a good long-term method for controlling rash.

Based on the results of the Skin Toxicity Evaluation Protocol with Panitumumab (STEPP) trial (see Table 2), preemptive therapy is more helpful than reactive therapy (Lacouture et al., 2010b). Application of a colloidal oatmeal lotion may ease symptoms and reduce inflammation. Antibiotic creams or gels may reduce symptoms of rash and seem to be well tolerated. Although sunscreen does not have an effect on rash severity, reducing sun exposure can limit the photosensitivity that may be enhanced with EGFR inhibitor therapy, which can add to the symptomatology seen with EGFR inhibitor–associated rash.

National guidelines do exist and are primarily based on recommendations from consensus panels of experts; these recommendations can guide clinicians in evidence-based therapy choices for rash and associated dermatologic side effects. The National Comprehensive Cancer Network (NCCN) has produced a consensus paper on the management of EGFR inhibitor–associated rash (Burtness et al., 2009), and MASCC has recently published care guidelines (Lacouture et al., 2011). Most guidelines are based on a comprehensive review of the literature; randomized clinical trials are the best source for the data used to develop recommendations for management of a particular treatment problem or disease state (see Table 3).

**Table 3:**
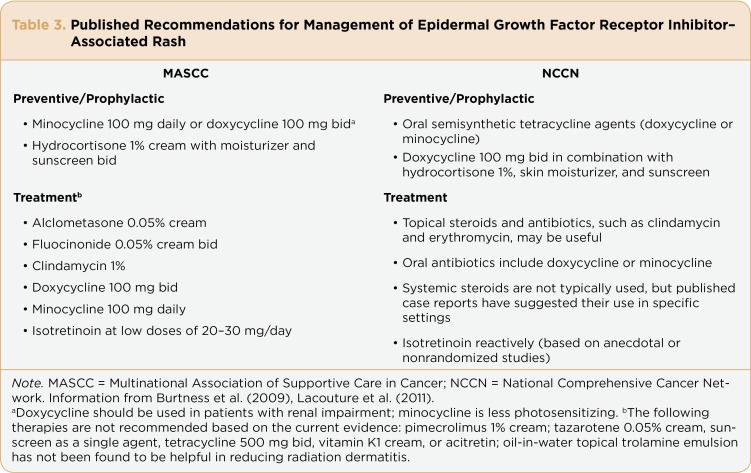
Table 3. Published Recommendations for Management of Epidermal Growth Factor Receptor Inhibitor–Associated Rash

The MASCC Skin Toxicity Study Group developed its recommendations based on levels of evidence: level I is reserved for meta-analyses of randomized clinical trials or randomized trials with high power; level II evidence represents randomized trials with lower power; level III evidence is from nonrandomized trials, such as cohort or case-controlled series; level IV includes descriptive and case studies; and level V evidence represents case reports and clinical examples (Lacouture et al., 2011). The NCCN recommendations are also based on an extensive review of published data on the management of EGFR inhibitor–associated rash. Consensus group recommendations have been published in the literature as well (Lynch et al., 2007).

It is worth noting that there is a financial impact associated with the diagnosis and treatment of dermatologic toxicities, including the rash associated with EGFR inhibitors. Borovicka and colleagues (2011) recently published the results of a single-center retrospective and prospective medical record data extraction. The study included 132 adult patients presenting between November 1, 2005, and June 30, 2008. All patients had been treated with a molecularly targeted agent. The main outcome measure was standard billable costs to the patient for dermatologic toxicity–related medications, clinic visits, laboratory and diagnostic testing, and therapeutic procedures. Significantly, the 132 patients had a median of 3 visits to the clinic for management of their dermatologic toxicities, which resulted in a median cost of $1,920 per patient. Although there were patients receiving agents other than EGFR inhibitors, the second most costly dermatologic toxicity was panitumumab-associated acneiform eruption, at a median cost of $933 per patient (*p* < .001; Borovicka et al., 2011).

Although skin rash is the most commonly seen dermatologic toxicity, there are several other toxicities worthy of discussion. Other forms of cutaneous toxicities include hair changes, radiation dermatitis enhancement, paronychia, pruritus/xerosis, and cracking and fissure development (Lacouture et al., 2011). In the paragraphs that follow we will focus on paronychia, pruritus/xerosis, and cracking/fissures, with discussion of optimal management strategies for each.

### Paronychia

**Figure 2:**
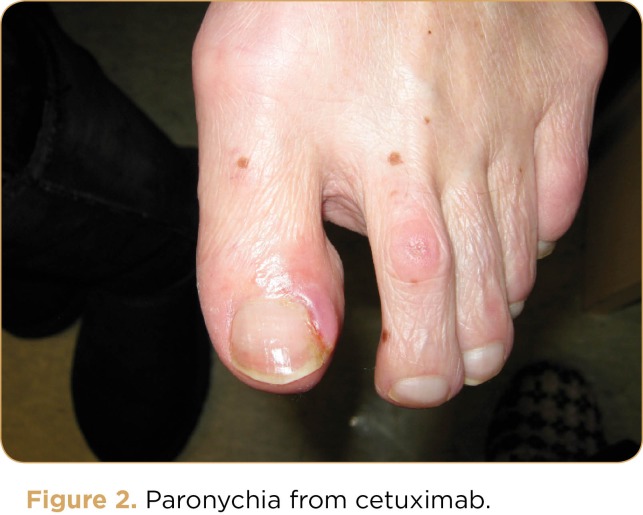
Figure 2. Paronychia from cetuximab.

Paronychia develops in approximately 10% to 15% of patients receiving EGFR inhibitor therapy (Ehmann, Ruzicka, & Wollenberg, 2011). The condition refers to the presence of tenderness, edema, or even purulent discharge in the nail folds (Lacouture et al., 2011); see Figure 2. Pyogenic granulomas may develop, requiring cauterization (Lacouture et al., 2011). Although it rarely leads to the need for cessation of therapy, this side effect can be painful, possibly contributing to a poorer quality of life (Ehmann, Ruzicka, & Wollenberg, 2011). Paronychia generally develops about 2 months after the initiation of therapy. Cultures of affected areas have demonstrated a myriad of bacterial infections as well as fungal infections, including *Candida albicans* (Ehmann, Ruzicka, & Wollenberg, 2011; Lacouture et al., 2011).

There are no randomized clinical trials specifically examining optimal treatment strategies for paronychia. The general recommendation is to apply topical antiseptic measures, administer diluted bleach soaks (a commonly used dilution contains one-quarter cup of bleach in three gallons of water [Burtness et al., 2009] ), and avoid irritating substances (Lacouture et al., 2011). Antibiotics such as the oral cephalosporins, tetracyclines, and fluoroquinolones may be employed if necessary (Lacouture et al., 2011; Ehmann, Ruzicka, & Wollenberg, 2011). Corticosteroids may be useful in reducing inflammation. Removal of the affected nail(s) may be required in certain cases.

### Pruritus/Xerosis

The EGFR inhibitor–associated rash can produce pruritus, which affects approximately half of all patients. Despite rarely requiring dose modifications or cessation of therapy, this symptom can negatively affect patients’ quality of life, interfering with sleep and other activities (Burtness et al., 2009). Pruritus can be present along with the EGFR inhibitor–associated rash; therefore, treatment of the rash can also help to relieve some of the accompanying pruritus (Lacouture et al., 2011). As itching often occurs subsequent to dryness and xerosis, strategies to prevent dryness are critical.

There are no published clinical studies specifically designed to examine optimal therapies for pruritus; case studies and anecdotal reports are the foundation for current recommendations. The MASCC guidelines recommend gentle skin care and the use of topical menthol 0.5% with pramoxine 1% and doxepin; medium- to high-potency steroids may be of help in relieving symptoms of pruritus (Lacouture et al., 2011). The use of topical agents such as moisturizing creams, specifically emollient creams that lack fragrances or irritants, has been suggested. Regenecare and Sarna Ultra have been recommended for the body, fluocinonide 0.05% cream or clobetasol foam may be helpful (Burtness et al., 2009; Eaby, Culkin, & Lacouture, 2008). Systemic treatment with antihistamines and gabapentin/pregabalin (Lyrica) has been recommended; the use of systemic doxepin may be effective in reducing itching in some patients (Lacouture et al., 2011).

Scalp rash is dermatologic in nature, though due to its location it is difficult to utilize the recommended topical creams and antibiotic gels. The oral semisynthetic tetracyclines do offer systemic relief and can help control the scalp rash or lesions. Over-the-counter shampoos containing selenium can be moisturizing for dry scalp. Prescription shampoos for dry scalp such as fluocinolone acetonide topical shampoo can penetrate to the scalp for symptom relief (Burtness et al., 2009).

### Cracking and Fissure Development

Cracking and fissure development is thought to occur as a result of the changes in keratinocyte differentiation during therapy with EGFR inhibitors. The stratum corneum deteriorates with a decrease in loricin, the protein keeping the epidermis intact (Lacouture et al., 2011). The skin’s characteristic tight basket-weave appearance is disrupted, leaving it loose with the epidermal layer unable to hold moisture; subsequently, the skin becomes particularly dry and can become inflamed (Lacouture et al., 2011).

Although there are no randomized clinical trials specifically examining therapies for cracking and fissure development, a number of anecdotal and case reports have recommended various treatment strategies. Patients should be educated on limiting the use of hot water, using mild moisturizing soaps or bath oils in an attempt to moisturize the skin, as well as wearing protective gloves and footwear (Lacouture et al., 2011). Bleach soaks may help to prevent infection. Zinc-containing creams can be helpful; patients can also use colloidal oatmeal bath products and creams in an effort to keep the skin hydrated (Alexandrescu, Vaillant, & Dasanu, 2007; Lacouture et al., 2011). In severe cases, medium- to high-potency steroid tape, hydrocolloid dressings, or topical antibiotics may be needed (Lacouture et al., 2011). Fissures often appear on the heels and fingertips; suggestions for treatment include the use of silver nitrate, aluminum chloride solution, zinc oxide, or a cyanoacrylate glue such as Krazy Glue (Burtness et al., 2009).

## Dermatologic Toxicities of Multikinase Inhibitors

The multikinase inhibitors are oral therapeutics that provide a novel approach to anticancer treatment by targeting multiple kinase pathways with a single drug. The typical adverse events are those that affect the skin, hair, nail beds, and mucosa. These can manifest as a papulopustular outbreak, erythematous rash, hand-foot skin reaction (HFSR), abnormally dry skin, pruritus, skin swelling, hyper-/hypopigmentation of the skin, and changes in the hair and around the nail beds. These dermatologic complications can occur in upward of 90% of patients receiving this therapy (Boers-Doets et al., 2011; Sternberg et al., 2010; Lacouture, Boerner, & LoRusso, 2006). Less common side effects including squamous cell carcinoma, keratoacanthoma, and eruptive melanocytic lesions have also been reported (Hong et al., 2008; Kong et al., 2007; Kong et al., 2008).

### Papulopustular and Erythematous Rash

The multikinase inhibitors that target EGFR have the same associated rash as was described earlier in this article. Interestingly, the majority of clinical trials that have been undertaken to ascertain an algorithm for prevention and management have been undertaken in patients receiving the monoclonal antibody EGFR inhibitors and have not routinely included patients receiving multikinase inhibitors. For patients receiving treatment with lapatinib (Tykerb), erlotinib, and vandetanib (Caprelsa), the erythematous papules and pustules predominantly affect seborrheic-rich areas of the face, scalp, and upper trunk. The lower trunk, extremities, and buttocks are less commonly affected (Boers-Doets et al., 2011). This rash can develop within the first few days through week 8 of treatment (Boers-Doets et al., 2011; Lee et al., 2009). For patients receiving multikinase inhibitors that do not directly target EGFR, this papulopustular rash can similarly occur, but it is less frequent and milder in severity (Rosenbaum et al., 2008; Lee et al., 2009).

Erythematous eruption on the face, scalp, and trunk has been described in patients receiving multikinase inhibitors that have several targets, such as sorafenib (Nexavar) and sunitinib (Sutent; Rosenbaum et al., 2008; Lee et al., 2009). These adverse events occur early with initiation of treatment and generally resolve within 6 to 8 weeks (Lee et al., 2009; McLellan & Kerr, 2011). The facial eruption appears like seborrheic dermatitis with erythema of the face, which can be aggravated by hot temperatures. There is no standard treatment for this rash. Therapies that have provided anecdotal relief include topical emollient of 2% ketoconazole or topical steroids (Robert et al., 2005). Patients can also experience scalp dysesthesia, which resolves in days to weeks (Autier, Escudier, Wechsler, Spatz, & Robert, 2008).

### Hair and Skin Discoloration

Hair depigmentation is associated with sunitinib. In phase I studies, patients developed alternating bands of hair depigmentation and pigmentation that corresponded to their cycles on and off treatment. One of the targets of sunitinib is stem cell factor receptor (Kit). Kit plays a role in the development and function of melanocytes, which are important to the maintenance of the hair follicle and proper pigment production. The hair changes seen with sunitinib are in essence a biological marker for the inhibition of Kit, which may also serve as a marker for efficacy (Moss et al., 2003). Alopecia can be seen to varying grades in patients receiving sorafenib or sunitinib. This hair loss can occur between weeks 3 and 15 of therapy; however, hair may begin to regrow while the patient is still on treatment (McLellan & Kerr, 2011). The new hair growth can be curly and brittle.

**Figure 3:**
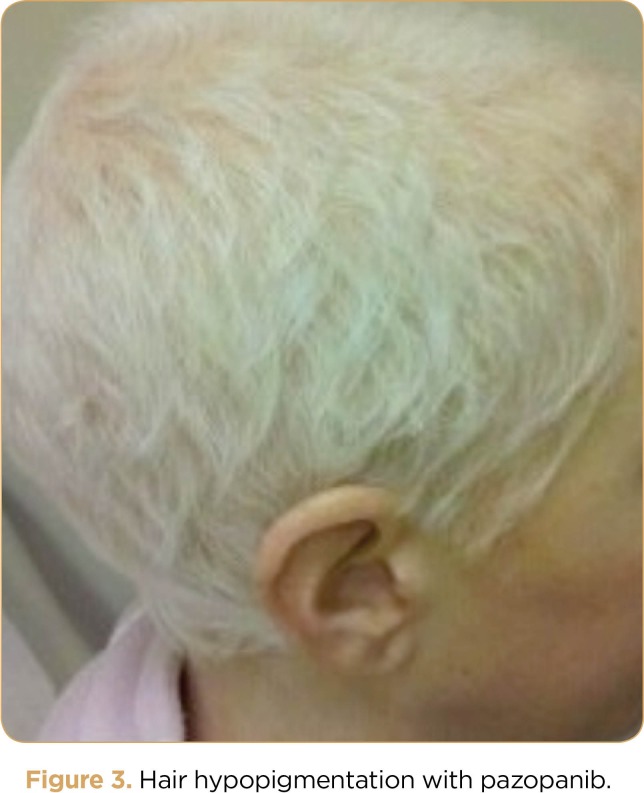
Figure 3. Hair hypopigmentation with pazopanib.

Yellow skin discoloration and hypopigmentation can also be caused by sunitinib. This can develop within the first week of treatment, and it generally resolves once treatment stops. Patients receiving pazopanib (Votrient) experienced skin hypo- or hyperpigmentation and hair hypopigmentation (Bible et al., 2010); see Figure 3. The mechanism for skin hyperpigmentation is unknown. As it can be aggravated by sun exposure, wearing a broad-spectrum sunscreen is strongly recommended.

### Hand-Foot Skin Reaction

**Figure 4:**
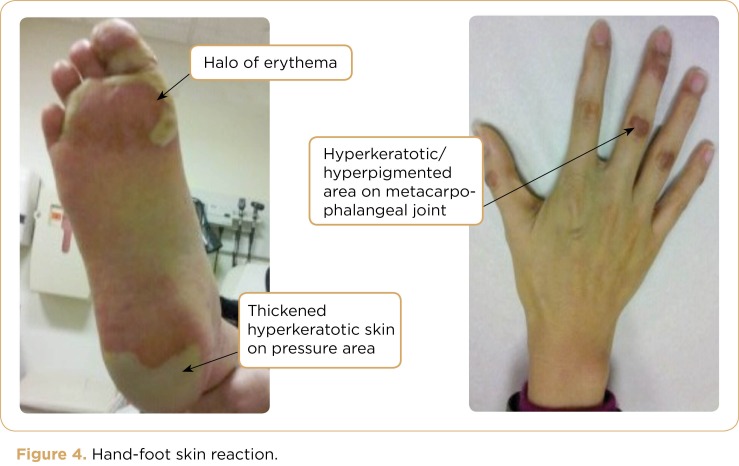
Figure 4. Hand-foot skin reaction.

A common dermatologic toxicity associated with multikinase inhibitors is hand-foot skin reaction (HFSR); see Figure 4. This can negatively impact patients’ quality of life, possibly leading to poor adherence, dose reductions, or discontinuation of potentially efficacious therapy. Hand-foot skin reaction has been shown to occur in anywhere from 9% to 62% of patients on multikinase inhibitors. It can develop within the first 2 to 4 weeks of treatment (Lacouture et al., 2008). Lesions can be tender and initially may appear with a peripheral halo of erythema localized at pressure areas (Porta, Paglino, Imarisio, & Bonomi, 2007). Similar developments can be identified on the distal phalanges and the fingertips, particularly around the nails (Yang et al., 2008). After several weeks, the lesions (with or without blisters) are followed by thickened, hyperkeratotic skin (calluses) that can be painful and impair activities of daily living and weight-bearing (Lacouture et al., 2008).

It should be noted that HFSR is different from and should not be confused with hand-foot syndrome (HFS), a cutaneous complication seen in patients receiving fluorouracil therapy, capecitabine (Xeloda), and liposomal doxorubicin. The similarities include erythema, blisters, and effects on the palms of the hands and soles of the feet. Hand-foot skin reaction also affects nonpressure areas of the finger webs and lateral feet and perianal skin. Additionally, HFSR can be accompanied by hyperkeratotic calluses. The typical pattern of localized hyperkeratotic lesions surrounded by erythematous areas distinguishes HFSR from HFS (Boers-Doets et al., 2011). Subjective symptoms of HFSR include paresthesia, burning, pain, and decreased tolerance to heat.

One of the proposed mechanisms responsible for the development of HFSR is drug leakage from capillaries in areas subject to repeated friction, grasping, or trauma. Another consideration for the pathogenesis is secretion of the multikinase inhibitor into the eccrine or sweat glands (Lacouture et al., 2008). The erythematous and tender nature of HFSR suggests that an inflammatory infiltrate may be present (Beldner et al., 2007).

At this time, there are no prospective, randomized trials to determine the best preventative and/or management strategies for HFSR. No evidence-based or clinical guidelines have been developed to date. Thus the approaches employed are solely based on anecdotal evidence. It is important to follow the prescribing information regarding the specific multikinase inhibitor and associated adverse events for dose reduction recommendations according to grade of severity.

Preventative recommendations include the removal of preexisting calluses and keratotic skin, use of orthotic devices to normalize weight-bearing and prevent friction, avoidance of friction and trauma to the hands and feet (especially during the first month of therapy) and use of thick cotton socks and gloves for protection of the hands and feet (Lacouture et al., 2008).

Management strategies for HFSR include use of agents such as urea 20%–40% cream and tazarotene 0.1% cream to hyperkeratotic areas; clobetasol propionate 0.05% ointment for erythematous areas and topical 2% lidocaine and/or systemic agents such as nonsteroidal anti-inflammatory drugs, pregabalin, and codeine for pain. Urea is a keratolytic that softens hyperkeratosis and decreases epidermal thickness. Tazarotene is a retinoid that decreases proliferation and reduces inflammation. These agents should be applied to affected areas no more than twice a day, as they can be irritating to unaffected skin (Lacouture et al., 2008).

The development of HFSR seems to be dose dependent with the multikinase inhibitors. The development of HFSR has not been correlated with response. Therefore, patients should not consider development of HFSR a sign of treatment efficacy.

## The Role of the Advanced Practitioner

Advanced practitioners (APs) manage patients receiving EGFR and multikinase inhibitor therapy in a variety of settings. They may be responsible for prescribing and/or administering EGFR and multikinase inhibitors; APs will also follow these patients during therapy to assess for and manage toxicities associated with these agents.

Evidence-based management strategies based on robust randomized clinical trials are preferred; strategies based on anecdotal reports or case studies are less preferable. Unfortunately, even the recommendations and guidelines for EGFR inhibitor–associated rash (MASCC, NCCN) are currently based on published data with limited numbers of patients. Larger trials are needed to provide clinically meaningful data that will clearly outline the most efficacious strategies for management of dermatologic toxicities associated with EGFR inhibitor therapies. Trials are also needed, specifically in patients receiving multikinase inhibitors, to assist in directing strategies for prevention and management of associated dermatologic adverse events with these agents, as many of the current trials focus on EGFR inhibitors. Until these trials are a reality, the AP should follow published guidelines when available and use clinical judgment in determining the usefulness of possible strategies based on case reports and anecdotal evidence.

As the use of EGFR and multikinase inhibitors increases, offering treatment benefits to patients diagnosed with a variety of tumor types, APs should anticipate dermatologic toxicities and treat patients in a proactive, evidence-based approach. As higher costs may be associated with patients suffering dermatologic toxicities, APs can be instrumental in the identification of optimal management strategies to improve patient outcome and possibly limit the financial burden of dermatologic adverse events. The role of supportive care often falls to the APs, and the oncology community may rely on APs to lead the way with randomized clinical trials looking at treatment strategies for EGFR and multikinase inhibitor therapy. It is incumbent upon APs to explain the importance of managing these toxicities to patients to improve enrollment in supportive care randomized clinical trials.
